# Maintenance of Hypoimmunogenic Features *via* Regulation of Endogenous Antigen Processing and Presentation Machinery

**DOI:** 10.3389/fbioe.2022.936584

**Published:** 2022-07-22

**Authors:** Ju-Hyun An, Hyebin Koh, Yujin Ahn, Jieun Kim, A-Reum Han, Ji Yoon Lee, Sun-Uk Kim, Jong-Hee Lee

**Affiliations:** ^1^ Futuristic Animal Resource and Research Center (FARRC), Korea Research Institute of Bioscience and Biotechnology (KRIBB), Ochang, South Korea; ^2^ Department of Functional Genomics, KRIBB School of Bioscience, Korea University of Science and Technology, Daejeon, South Korea; ^3^ Department of Medical Life Sciences, College of Medicine, The Catholic University of Korea, Seoul, South Korea; ^4^ National Primate Research Center (NPRC), Korea Research Institute of Bioscience and Biotechnology (KRIBB), Ochang, South Korea; ^5^ CHA Advanced Research Institute, Bundang CHA Hospital, CHA University, Seongnam, South Korea

**Keywords:** hypoimmunogenic cell, universal donor cell, regenerative cell therapy, antigen presentation and processing machinery, cell therapy

## Abstract

Universally acceptable donor cells have been developed to address the unmet need for immunotypically matched materials for regenerative medicine. Since forced expression of hypoimmunogenic genes represses the immune response, we established universal pluripotent stem cells (PSCs) by replacing endogenous β2-microglobulin (β2m) with β2m directly conjugated to human leukocyte antigen (HLA)-G, thereby simultaneously suppressing HLA-I expression and the natural killer (NK) cell-mediated immune response. These modified human PSCs retained their pluripotency and differentiation capacity; however, surface presentation of HLA-G was absent from subsequently differentiated cells, particularly cells of neural lineages, due to the downregulation of antigen processing and presentation machinery (APM) genes. Induction of APM genes by overexpression of NLR-family CARD domain-containing 5 (NLRC5) or activator subunit of nuclear factor kappa B (NF-κB) heterodimer (RelA) recovered the surface expression of HLA-G and the hypoimmunogenicity of neural cells. Our findings enhance the utility of hypoimmunogenic cells as universal donors and will contribute to the development of off-the-shelf stem-cell therapeutics.

## Introduction

The unlimited self-renewal and differentiation potentials in all cell lineages of pluripotent stem cells (PSCs) make them valuable cell sources for regenerative medicine. The allogeneic transplantation of PSCs or their differentiated derivatives is a promising therapeutic approach for several incurable diseases. However, human leukocyte antigen (HLA) incompatibility between donor and recipient restricts the coverage of cell-replacement therapy. Together with advances in gene-editing technologies, there have been several attempts to generate hypoimmunogenic cells using PSCs compatible with any recipient.

To evade the innate immune response, HLA class I (HLA-I) molecules on the cell surface must be knocked out. The first generation of hypoimmunogenic cells used various HLA-I knock-out (KO) strategies to protect PSCs against CD8^+^ cytotoxic T (Tc) cells (Lu et al., 2013; Riolobos et al., 2013; Karabekian et al., 2015). However, the loss of HLA-I stimulates natural killer (NK) cell-mediated immune responses (Wang et al., 2015; Das et al., 2017), leading to the failure of host innate immune evasion. To address this, the second generation of hypoimmunogenic cells was created by gene editing to express immunomodulatory genes, such as the non-classical HLA-I molecules HLA-E (Gornalusse et al., 2017) and HLA-G (Han et al., 2019). These cells lack immune-stimulatory ligands and overexpress immunomodulatory genes, enabling evasion of host immunity. However, the expression of these potent repressors of NK and T-cells could be epigenetically restricted in cells of certain lineages (Cabrera et al., 2007; Suarez-Alvarez et al., 2010), leading to the absence of immunomodulatory activity in gene-edited cells.

The marginal expression of HLA-I genes in neural stem and progenitor cells (NPCs) has been investigated (Liu et al., 2013) and is assumed to enable NPCs to evade host innate immunity during allogeneic transplantation therapy (Bonnamain et al., 2012). For this reason, the allogeneic transplantation of NPCs has therapeutic potential for incurable neurological diseases, such as Parkinson's disease, Alzheimer's disease, and amyotrophic lateral sclerosis. Although the allogeneic transplantation of NPCs was tolerable in some cases regardless of immunosuppression (Fischer et al., 2020), transplanted NPCs activated the host immune system and NK cells, leading to graft rejection in mice (Phillips et al., 2013). Furthermore, in humans, induced pluripotent stem cell (iPSC)-derived NPCs and dopaminergic neurons were vulnerable to allogeneic NK cell-mediated immune reaction but were protected by interferon-gamma (IFN-γ) treatment, which upregulated surface HLA-I expression (de Rham et al., 2020).

Here, we constructed a hypoimmunogenic cell line by editing the β2m gene in human iPSCs (hiPSCs) using CRISPR/Cas9 technology. We knocked-in the immunomodulatory gene, HLA-G, fused with β2m into β2m loci of hiPSCs, resulting in immunogenicity loss and immunomodulatory activity gain. This hypoimmunogenic cell line was differentiated into hematopoietic- and neural-lineage cells. As expected, neural-lineage cells under normal physiological conditions did not express immune-inhibitory HLA-G on the cell surface, although its expression was controlled by a constitutive promoter. This transgene silencing was rescued by IFN-γ, which significantly upregulated HLA-G surface presentation. We investigated this phenomenon using several methods; we found that APM genes modulate pan-HLA-I surface presentation in neural cells and are responsible for transgene silencing. However, because gene-edited cells cannot be stimulated with IFN-γ in a therapeutic context, we sought a method of modulating HLA expression in a cell-autonomous manner. HLA-G surface presentation was recovered *via* transfection of transcription factors responsible for APM gene regulation. Transfection of those transcription factors resulted in HLA surface presentation in differentiated NPCs. Consequently, HLA-G-recovered NPCs were protected from NK cell-mediated immune responses. These findings are expected to enhance the therapeutic utility of hypoimmunogenic cells for all human tissues, irrespective of their epigenetic background.

## Materials and Methods

### Cell Culture

The human-induced pluripotent stem cells (hiPSCs) were cultured on matrigel (BD Biosciences, Franklin Lakes, NJ, United States)-coated plates with TeSR-E8 culture medium (Stemcell Technologies) as previously described (Choi et al., 2019). Cells were passaged using ReLeSR (StemCell Technologies) at a ratio of 1:6 every 5–6 days. The pluripotency of the iPS cell line was validated using *in vivo* teratoma assay and confirmed to have full differentiation potential for three germ layers (Supplementary Figure S2). NK92 and K562 cells were purchased from the American Type Culture Collection (ATCC) and maintained in accordance with the manufacturer’s instructions.

### Differentiation Into Hematopoietic and Neural Lineages

For hematopoietic differentiation, the StemDiff™ Hematopoietic Kit (Stem Cell Technologies) was used in accordance with the manufacturer’s instructions. For neural differentiation, iPSCs were dissociated into single cells using Accutase™ (Stem Cell Technologies), transferred at 2,000 cells/well into 96-multiwell ultra-low attachment (ULA) round-bottom plates (BD Falcon), and centrifuged for 5 min at 1,500 rpm for embryoid body formation. Next, neural induction was performed in three different types of media. Embryoid bodies were incubated for 3 days in Dulbecco’s modified Eagle medium (DMEM)/F12 (Thermo Fisher) supplemented with N2 (Themo Fisher), B27 (Thermo Fisher), 100 nM LDN193189 (Tocris) and 10 μM SB431542 (Tocris). Subsequently, 20 ng/ml rhFGF basic (R&D Systems) and 20 ng/ml rhEGF (R&D Systems) were added. After incubation for a few days, EBs were plated onto poly-L-ornithine (Sigma) and L-laminin (Thermo Fisher)-coated plates and then neural rosettes were manually collected and maintained in DMEM/F12 supplemented with N2, B27, 20 ng/ml rhFGF basic, 20 ng/ml rhEGF, and 3 μM CHIR99021 (Tocris).

### *In Vivo* Teratoma Formation Assay

hiPSCs for teratoma formation assays were grown in TeSR-E8 culture medium (Stemcell Technologies) on vitronectin-coated plates (Stemcell Technologies). The cells were dissociated with dispase (Stemcell Technologies) and triturated as small clumps and injected into testis sites (∼1 × 10^6^ cells per site) of three 8-week-old male NOD. Cg-Prkdc^scid^IL2^tmlWjl^/Szj mice (NOD-SCID IL-2rg null, The Jackson Laboratory). Tumor formation was observed in the mouse and harvested on day 120 post-injection. The tumor samples were fixed in 4% paraformaldehyde, embedded in paraffin, cut into 5 μm serial sections, stained with hematoxylin and eosin (H&E) for detection of teratoma, and photographed for histological analyses using Zeiss axionscan microscopy (Axio Imager. Z.1, Carl Zeiss).

### Plasmid Construction and Genome Editing

Complementary DNAs (cDNAs) of human genes were subcloned from a cDNA library that had been created using Human Total RNA Control (Thermo Fisher). cDNA of NLRC5 was subcloned from myc-NLRC5 from Addgene (plasmid #37509); NLRC5mut was generated by polymerase chain reaction (PCR)-based point-directed mutagenesis. Homology arms for β2m were amplified from iPSC genomic DNA (gDNA) by conventional PCR. Each component was assembled by PCR amplification using the InFusion® HD Cloning Kit (Clontech). For gene editing, guide RNA (gRNA) was designed using an online CRISPR design tool (Zhang Lab); predicted exonic off-target sites were listed and sequenced after cell lines had been established. gRNA sequence and primers for off-target sequencing are listed in Supplementary Table S1. Donor templates for homologous recombination (HR) and CRISPR/Cas9 plasmids (Addgene plasmid #79144) were transfected into iPSCs using the P3 Primary Cell 4D-Nucleofector Kit (Lonza), in accordance with the manufacturer’s instructions. Cells were next seeded onto Matrigel^®^-coated plates with 10 μM Y27632 (Tocris). Cells were selected using 500 μg/ml Geneticin™ Selective Antibiotic (Thermo Fisher) for >2 weeks. The remaining single-cell colonies were transferred to Matrigel^®^-coated 24-multiwell plates and expanded. Each colony’s gDNA was isolated and subjected to PCR for genotype analysis. To establish an EGFP cell line, β2m-HLA-G in a donor template was replaced with EGFP, using the procedures implemented for iPSC gene editing.

### DNA Isolation and Genotyping

gDNA was isolated using the DNeasy Blood & Tissue Kit (Qiagen), in accordance with the manufacturer’s instructions. To genotype the target site, primers flanking the gene-editing site were designed; these yielded 385-bp and 5-kb products from the wild-type (WT) and transgene-integrated chromosomes, respectively. gDNA was amplified using a previously described primer set and KOD One™ PCR Master Mix (Toyobo). The products were visualized by agarose gel electrophoresis with ethidium bromide (Sigma). A 1-kb DNA ladder (Thermo Fisher) was used to measure the size of amplified DNA. Gels were imaged using the GelDoc System (Bio-Rad).

### RNA Isolation and Reverse Transcription Quantitative PCR

Total RNA was isolated using the RNeasy Kit (Qiagen), in accordance with the manufacturer’s instructions. cDNA was synthesized using the SuperScript™ III First-Strand Synthesis System (Thermo Fisher) with random hexamer primers. For quantification of transcript levels, RT-qPCR was performed using SYBR™ Green PCR Master Mix (Applied Biosystems™) and the StepOnePlus™ Real-Time PCR System (Applied Biosystems™). Amplification was performed as follows: 95°C for 10 min, followed by 45 cycles of 95°C for 15 s and 60°C for 1 min. The primer sets used for RT-qPCR are listed in Supplementary Table S1. Relative expression levels of target genes were analyzed by the 2_t_^−ΔΔC^ method (Lee et al., 2014) and normalized to the housekeeping gene, GAPDH.

### Immunocytochemistry

Cells were plated on 24-multiwell plates, then fixed and permeabilized using the Fixation/Permeabilization Solution Kit (BD), in accordance with the manufacturer’s instructions. Next, antibodies diluted in Perm/Wash buffer (BD) were added and then samples were incubated with antibodies overnight at 4°C. Nuclei were counter-stained with Hoechst 33342 (10 μg/ml, Sigma) for 15 min at room temperature. Immunofluorescence images were obtained using a fluorescence microscope (Leica) and confocal images were obtained using a confocal microscope (Zeiss). For staining, Oct3/4 (AlexaFluor488, 1:50, BD Pharmingen), Nanog (PE, 1:50, BD Pharmingen), Sox2 (AlexaFluor555, 1:50, BD Bioscience), Tra-1-60 (AlexaFluor488, 1:100, BD Pharmingen), SSEA-3 (PE, 1:100, BD Pharmingen), SSEA-4 (AlexaFluor488, 1:100, BD Bioscience), Tuj1 (AlexaFluor488, 1:100, BD Bioscience), Nestin (AlexaFluor488, 1:100, Millipore), CD107a (APC, 1:100, Biolegend), HLA-G (PE, 1:100, Abcam), HLA-BC (APC, 1:100, eBioscience), human β2m (Abcam), and goat anti-mouse IgG secondary (AlexaFluor555, Thermo Fisher) antibodies were used.

### Flow Cytometry

Cells were dissociated into single cells using Cell Dissociation Buffer (Gibco) and stained with fluorochrome-conjugated antibodies. In detail, cells were incubated with Cell Dissociation Buffer for 15 min at 37°C and resuspended in DPBS containing 3% FBS. Cells were centrifuged for 5 min at 1,200 rpm, then distributed to 5-ml round-bottom polystyrene tubes (Falcon). Dead cells were stained with the Fixable Near-IR Dead Cell Stain Kit (APC-Cy7, 1:7500, Thermo Fisher) or Fixable Viability Stain 575V Kit (1:1,000, BD Bioscience) for 30 min at 4°C, then washed. Antibodies were added at the appropriate concentrations and incubated for 30 min at 4°C. For intracellular protein staining, cells were fixed in Fixation Buffer (BD Bioscience) and stained with antibodies diluted in Perm/Wash Buffer. After staining, cells were washed and analyzed using a FACSAria™ III (BD Bioscience). Data were analyzed by FlowJo software (Tree Star). For staining, in addition to the antibodies used for immunocytochemistry, CD56 (PE, 1:100, Biolegend), CD34 (FITC, 1:100, BD Bioscience), and CD45 (FITC, PE-Cy7, 1:100, BD Bioscience) antibodies were used.

### Western Blotting

Samples were lysed in protein lysis buffer [RIPA buffer (Elpis), PhosSTOP (Roche), and protease inhibitor (Sigma)]. Protein was quantified using a Pierce™ BCA Protein Assay Kit (Thermo Fisher). Protein samples were separated by sodium dodecyl sulfate-polyacrylamide gel electrophoresis and transferred to nitrocellulose membranes (Bio-Rad). After they had been blocked with 5% skim milk (Sigma) in Tris-buffered saline plus Tween (TBST), blots were incubated with a mouse-human β2m (1:1000) or human GAPDH (1:1000, Cell Signaling Technology) antibody overnight at 4°C. Blots were next washed with TBST and incubated with the goat anti-mouse IgG HRP-conjugated secondary antibody (1:5000, Cell Signaling Technology) in 5% skim milk in TBST for 2 h at room temperature with agitation. Blots were washed, incubated with ECL solution (Pierce™ ECL Western Blotting Substrate kit, Thermo Fisher), and imaged using the ChemiDoc System (Bio-Rad).

### Natural Killer Cell Cytotoxicity Assay

NK cell-specific cytotoxicity was assessed by NK cell degranulation assays and calcein acetoxymethyl ester (calcein-AM) release assays. For NK cell degranulation assays, NK cells were co-cultured with target cells at a 1:1 ratio for 4 h at 37°C in the presence of CD107a-APC antibody in a 96-multiwell ULA round-bottom plate. After 1 h, 10 mg/ml Brefeldin A (Sigma) was added and retained in the medium for the last 3 h of co-culture. Next, cells were washed and reacted with a CD56-PE antibody. Samples were transferred to 5-ml polystyrene tubes, washed with DPBS containing 3% FBS, and analyzed using the BD FACSAria™ III. For calcein-AM release assays, 1 × 10^6^ target cells were labelled with calcein-AM (10 μM, Thermo Fisher) for 30 min at 37°C with intermittent shaking protected from light. After calcein-AM labeling, cells were diluted to 5 × 10^3^ cells per 100 μl of culture medium and transferred to 96-multiwell ULA round-bottom plates. NK cells were enumerated and serially diluted to the desired effector-to-target (E:T) ratio. NK cells were next transferred to 96-well ULA plates, except for the minimal- and maximal-release control groups, which were transferred to the culture medium and 2% Triton X-100 (Sigma), respectively. Cells were co-cultured for 4 h at 37°C, then centrifuged at 1,000 rpm for 5 min; 75 μl of supernatant were transferred to a 96-well assay plate (Corning). Fluorescence was measured on a SpectraMax iD3 microplate reader (Molecular Devices) at a wavelength of 488 nm. The specific cell lysis was calculated by the following equation:

$$Specific\;lysis\;\left(\%\right)=\frac{Sample\;release-Minimal\;release}{Maximal\;release-Minimal\;release}\times 100.$$

### Statistical Analyses

Data are shown as means and standard deviations. Experiments were performed in (at least) triplicate. Statistical significance was evaluated using GraphPad Prism and Microsoft Excel softwares, according to unpaired Student’s *t*-test analyses. A value of *p* < 0.05 was considered indicative of statistical significance (*p < 0.05, **p < 0.01, and ***p < 0.001).

## Results

### Construction of HLA-G-Overexpressing K562 Cells

**FIGURE 1:**
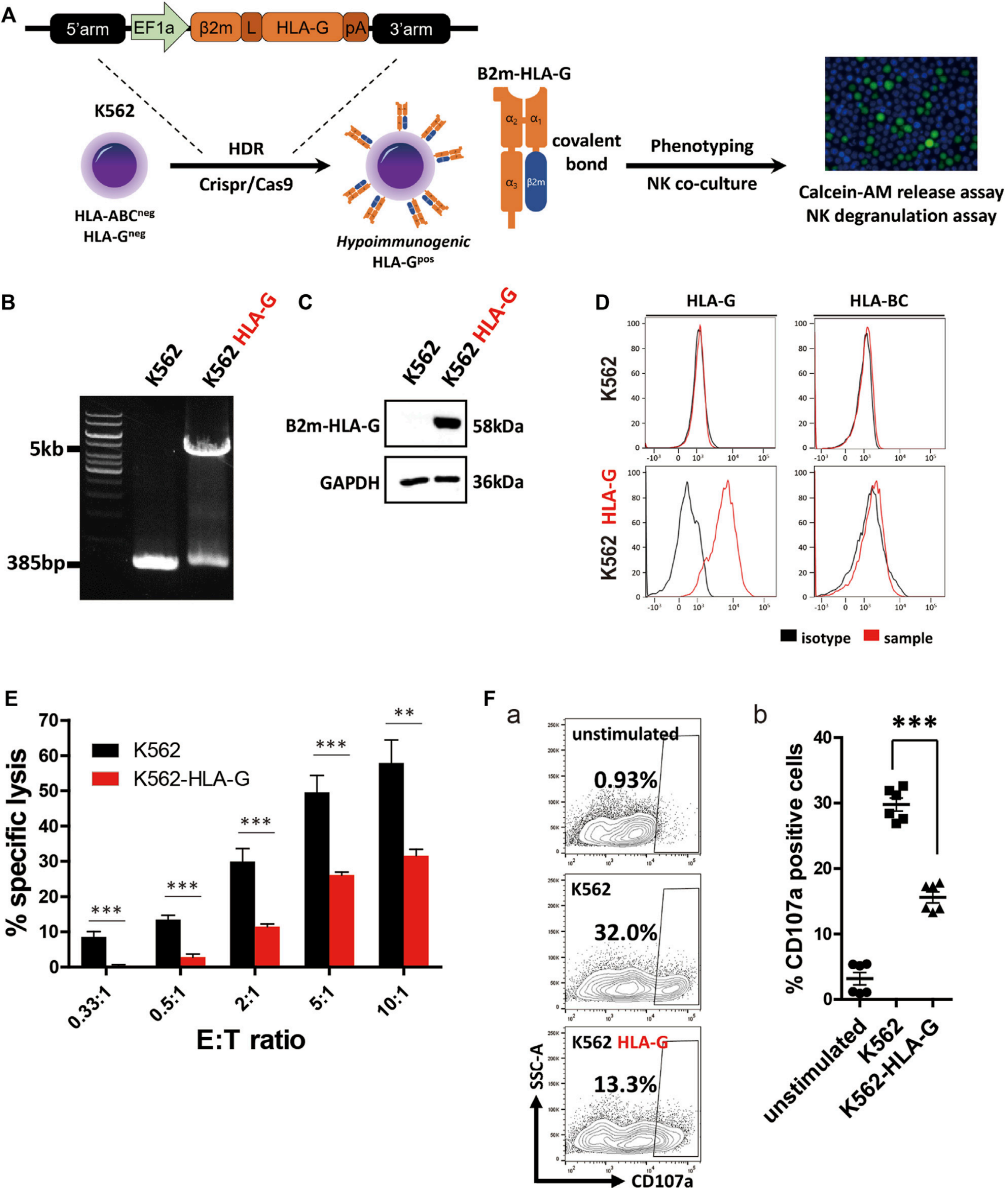
Generation of an HLA-G-overexpressing K562 cell line (K562-HLA-G) and evaluation of its hypoimmunogenic activity. **(A)** Schematic of the generation of the hypoimmunogenic K562 cell line. **(B)** Genotyping of the HLA-G over-expressing K562 cell line by conventional PCR. **(C)** Covalently bound β2m and HLA-G were confirmed by western blotting **(D)** Surface presentation of HLA-BC and HLA-G by flow cytometry **(E)** NK92 cells were less cytotoxic to K562-HLA-G cells than to WT K562 cells, according to calcein-AM release assays (*n* = 4). **(F)** K562-HLA-G cells repressed NK degranulation. **(a)** NK cell degranulation at the indicated target cells. NK cell degranulation marker (CD107a)-positive cells were gated as shown in Panel **(F)**. **(b)** CD107a-positive populations (*n* = 6). Data are means ± standard errors of the mean (SEMs). *p < 0.05, **p < 0.01, ***p < 0.001.

HLA-G, a non-classical HLA-I molecule, promotes maternal-fetal immune tolerance by inhibiting immune cells, including T, NK, and dendritic cells (Hunt et al., 2005). In cancer, the expression of soluble and membrane HLA-G isotypes is a marker of a poor prognosis (Krijgsman et al., 2020). To confirm the immune-inhibitory potential of HLA-G, we constructed HLA-G-overexpressing K562 cells; this human erythroleukemia cell line lacks HLA-I expression and is thus highly vulnerable to NK cells. To present HLA-G on the cell surface, cDNAs of HLA-G and β2m were fused with a flexible linker (Supplementary Figure S1; Figure 1A). To establish a stable cell line, we constructed an HR donor template using a β2m-HLA-G fusion construct, then created a CRISPR/Cas9 all-in-one plasmid targeting the first exon of β2m. After transfection and antibiotic selection, gDNA was isolated and amplified using a specific primer set (Supplementary Table S1). The cells harbored a 5-kb transgene (Figure 1B), and surface expression was confirmed by flow cytometry. WT K562 cells did not express HLA-G or classical HLA-I molecules (HLA-BC), whereas HLA-G-knock-in K562 cells (K562 HLA-G) showed HLA-G expression on the surface (Figure 1D). Western blotting was then performed to examine the structure of artificially fused β2m and HLA-G. A 58-kDa β2m-HLA-G protein was detected, which was the expected combination of 11-kDa β2m and 45-kDa HLA-G (Figure 1C). Therefore, our fusion construct was stably expressed and properly translocated.

### Hypoimmunogenic Potential of HLA-G

To assess the hypoimmunogenic potential of HLA-G, we performed calcein-AM release assays and NK cell degranulation assays using K562 and NK92 cells as effector cells. NK cell-mediated target cell-specific lysis increased with increasing E:T ratio (Figure 1E). At E:T ratios of 0.33:1 to 10:1, mean specific lysis percentages in control cells were 8.53%, 13.4%, 29.87%, 49.54%, and 57.87%, respectively. In contrast, K562-HLA-G cells showed significantly lower cytotoxicity (0.36%, 2.81%, 11.48%, 26.12%, and 31.59%, respectively) than did control cells at all E:T ratios.

NK cell degranulation assays evaluate NK cell activation by detecting the granule membrane protein marker CD107a (LAMP1) on the plasma membrane. After co-culture with target cells, surface CD107a and the NK cell marker CD56 were reacted with fluorescently labeled antibodies. K562-HLA-G cells showed significantly lower NK cell degranulation than did WT K562 cells (Figures 1F,a). The mean activation of NK cells co-cultured with K562-HLA-G was 15.6%, compared to 29.7% for NK cells co-cultured with WT K562 cells (Figures 1F,b). Therefore, ectopic expression of HLA-G in HLA-I-null cells suppressed NK cell-mediated immune responses.

### Generation of Hypoimmunogenic Human iPSCs

**FIGURE 2:**
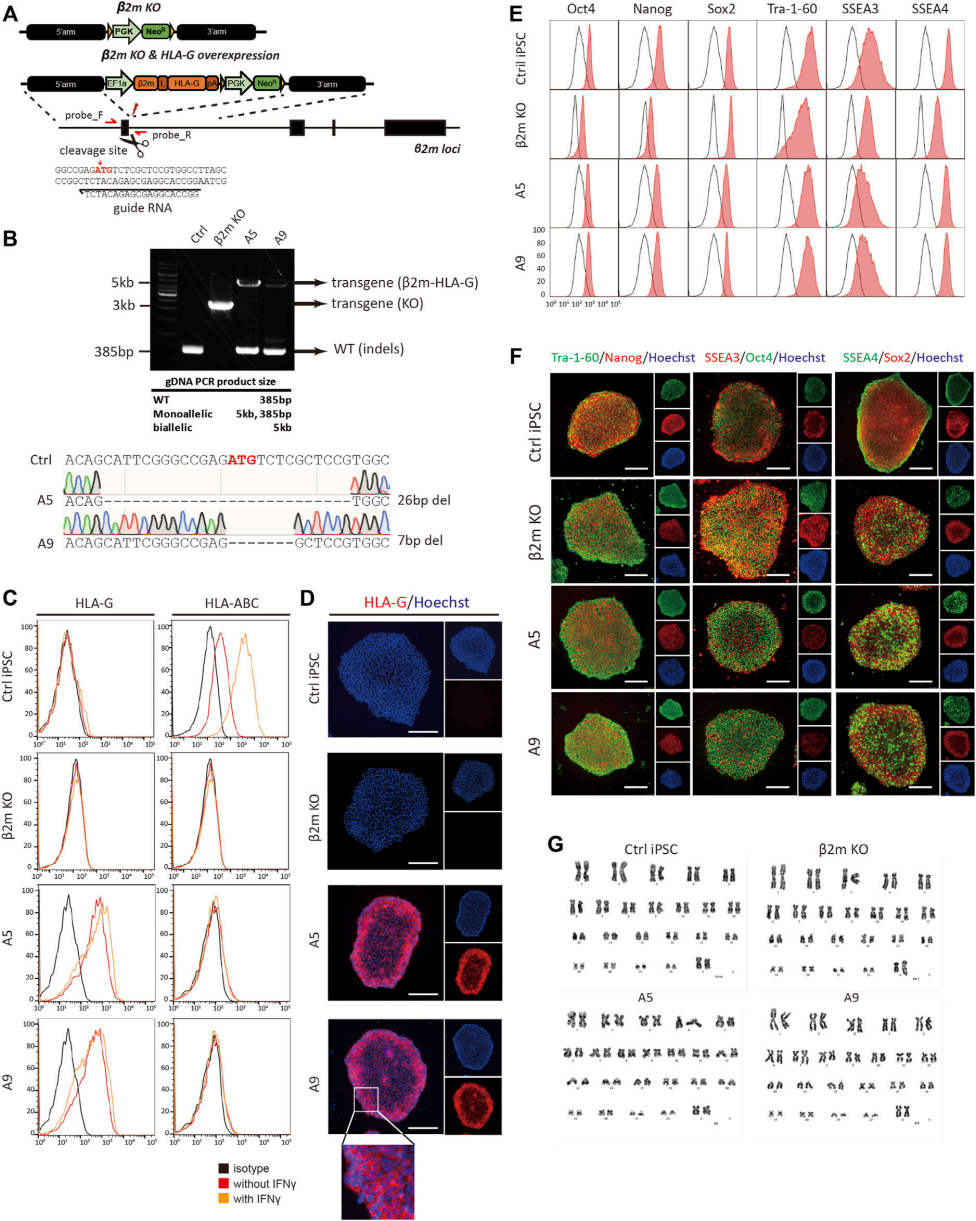
Generation and characterization of hypoimmunogenic cell lines. **(A)** Schematic of the donor plasmid for homology-directed repair and the gRNA sequence used for gene editing. **(B)** Genotyping of cell lines by conventional PCR. Indels at target loci were sequenced by the Sanger method. **(C)** Flow cytometric analysis of surface phenotype. HLA-G and HLA-BC expression patterns with (orange) or without (red) IFN-γ in control (Ctrl iPSC), β2m knock-out (β2m-KO), and HLA-G-overexpressing cell lines (A5 and A9) and the isotype control (black). **(D)** Immunocytochemistry of surface HLA-G expression; all images are fluorescence micrographs. **(E,F)** Characterization of pluripotency markers by flow cytometry and immunocytochemistry. **(G)** Karyotyping by GC-banding. Scale bar, 250 μm.

We next created hypoimmunogenic cells with HLA-G using hiPSCs, and confirmed three germ layers differentiation potential *in vivo* teratoma assay (Supplementary Figure S2). Donor templates for HR and CRISPR/Cas9 plasmids were used to generate hypoimmunogenic hiPSCs (Figure 2A). hiPSCs were transfected, then selected using antibiotics. gDNA was isolated from single-cell colonies; the target locus was amplified by conventional PCR with the primers in Supplementary Table S1. To ensure the complete KO of β2m by CRISPR/Cas9 induced HR in iPSCs, western blot, immunocytochemistry, and flow cytometry were performed. The β2m KO cell lines were generated by the previously described strategy and identified by gDNA PCR and sequencing. We found that there was no β2m expression or surface presentation in every β2m KO cell line (Supplementary Figure S3). After that, we changed the HR donor and conducted the same procedures for making hypoimmunogenic cells. We selected two hypoimmunogenic cell lines, A5 and A9, for further experiments. Although those cell lines were heterozygous, gDNA sequencing revealed that the non-transgene strand in A5 and A9 cell lines had 26-bp and 7-bp indels, respectively. These indel mutations deleted the start codon of β2m and caused a frameshift mutation, resulting in complete KO of surface HLA-I expression (Figure 2B).

To detect the expression of the β2m-HLA-G fusion construct and classical HLA-I molecules, flow cytometry and immunocytochemistry were performed using fluorescently labeled anti-HLA-G and -HLA-BC antibodies (Figures 2C,D). WT hiPSCs expressed HLA-BC on the surface under normal physiological conditions; this expression was upregulated in response to 48 h of treatment with 25 ng/ml IFN-γ. In contrast, β2m KO, A5, and A9 cell lines did not show surface expression of HLA-BC, regardless of IFN-γ treatment (Figure 2C, right panel). β2m-HLA-G, the fusion construct, was expressed in A5 and A9 cells but not in WT and β2m KO cells (Figure 2C, left panel). This surface phenotype was confirmed by immunocytochemistry (Figure 2D). Immunofluorescence images showed HLA-G on the surfaces of A5 and A9 cells (Figure 2D, magnified image).

We evaluated the pluripotency and genomic stability of gene-edited cell lines. All cell lines expressed the pluripotency markers Oct4, Nanog, Sox2, Tra-1-60, SSEA3, and SSEA4, as determined by flow cytometry (Figure 2E) and immunocytochemistry (Figure 2F). All gene-edited cell lines showed a normal karyotype, 44 + XX, as did the WT iPSC line (Figure 2G). To investigate whether gene editing caused unwanted mutations, the cells were subjected to off-target analysis. All predicted exonic off-target candidate sites were PCR-amplified and sequenced (Supplementary Figure S4). The above findings show that we created hypoimmunogenic cell lines of reliable HLA phenotypes, which were not perturbed under inflammatory conditions.

### Human Leukocyte Antigen Phenotype of Cell Lines Differentiated From Hypoimmunogenic iPSC Lines

We differentiated the cells into hematopoietic and neural lineages, which have therapeutic potential for incurable diseases. An immunosuppressant regimen is indispensable for the transplantation of both lineages, but their immunogenic phenotypes differ. Whereas NSCs and their derivatives show marginal expression of HLA and have low immunogenicity (Johansson et al., 2008), hematopoietic stem cells exhibit high HLA expression (Gornalusse et al., 2017).

**FIGURE 3:**
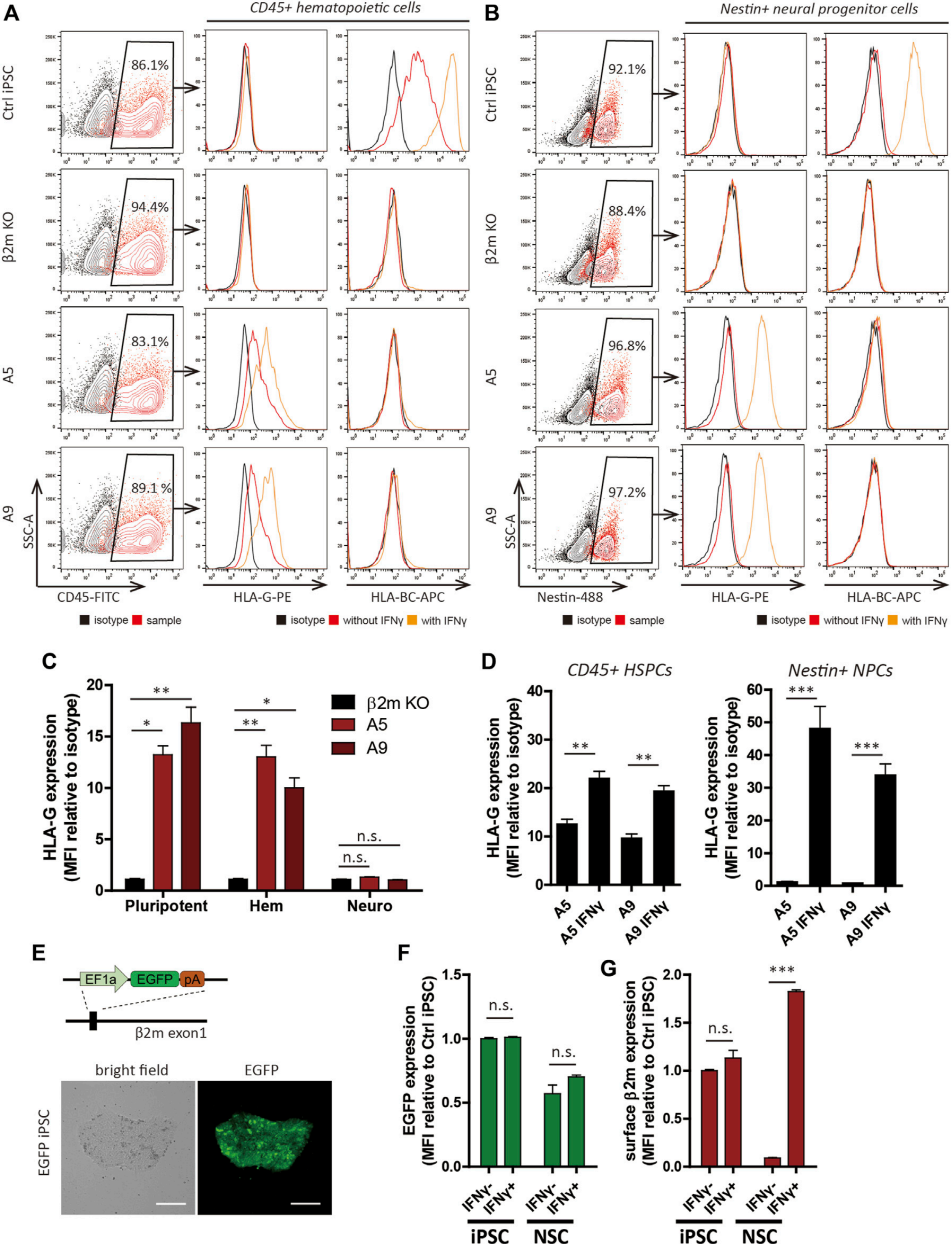
Differentiation and HLA expression profiles of hypoimmunogenic cell lines. **(A,B)** Characterization of CD45^+^ hematopoietic progenitors **(A)** and nestin^+^ neural progenitor cells **(B)** derived from the cell lines. HLA-G and HLA-BC expression patterns with (orange) or without (red) IFN-γ, determined by flow cytometry. **(C,D)** Relative MFI of HLA-G compared to the isotype control (*n* = 3). MFI of pluripotent cells (pluripotent), CD45^+^ hematopoietic lineage cells (Hem), Nestin^+^ neural lineage cells (Neuro) under control (**(C)**, *n* = 3) and IFN-γ-treated (**(D)**, *n* = 4) conditions. **(E)** Schematic of generation of the EGFP cell line (upper) and light and fluorescence micrographs. Scale bar, 250 μm. **(F,G)** EGFP expression patterns in EGFP cells **(F)** and surface β2m expression patterns in WT cells **(G)** of iPSCs and NSCs with or without IFN-γ by flow cytometry (*n* = 3). Data are means ± SEMs. N.s., not significant. *p < 0.05, **p < 0.01, ***p < 0.001.

After 15 days of hematopoietic differentiation, the resulting round, floating cells were presumed to be blood cells (Supplementary Figure S5A). According to flow cytometry analyses, >80% of the cells expressed CD45 (Figure 3A) and >40% expressed both CD45 and CD34 (Supplementary Figure S5D). Flow cytometry of differentiated NSCs (Supplementary Figure S5A) showed that most expressed the neural progenitor cell marker, nestin (Figure 3B). Furthermore, the neural stem cell markers Sox2 and nestin were detected by immunocytochemistry in the nucleus and cytoplasm, respectively, of differentiated cells (Supplementary Figure S5B). The differentiation potentials of those cells were demonstrated *via* differentiation into neuronal cells, which showed morphological changes and Tuj1-positive exon projections (Supplementary Figure S5C).

Next, we investigated the surface HLA phenotype by flow cytometry. As expected, the HLA phenotype trends in HPCs were identical to the HLA phenotype trends in pluripotent cells (Figure 3A). Surface HLA-BC expression was detected in WT cells but not A5 and A9 cells. HLA-G was detected in A5 and A9 cell-derived HPCs but not in WT cells. NSCs derived from the cell lines showed expression trends distinct from the trends of pluripotent cells or hematopoietic-lineage cells (Figure 3C). As previously reported (Mehler et al., 2020), HLA-I expression was diminished after neural differentiation and recovered by IFN-γ treatment; however, this pattern was not observed in the gene-edited cell lines (Figure 3B). HLA-G expression in the gene-edited cell lines was perturbed after differentiation, although it was controlled by a constitutive promoter. The diminished surface expression of HLA-G was retained after IFN-γ treatment (Figures 3C,D). HLA-I and HLA-G expression levels gradually diminished in response to the withdrawal of IFN-γ (Supplementary Figure S6) in control and gene-edited cell lines, respectively.

Because the transcriptional activity of a constitutive promoter can differ among cell types (Qin et al., 2010) or chromosomal landscape of the site of integration in relation to epigenetic factors (Williams et al., 2008), we created EGFP-expressing cell lines, which have a genetic structure identical to hypoimmunogenic cells (Figure 3E; Supplementary Figure S7A); flow cytometry analyses of these cell lines enabled measurement of the transcriptional activity of EF1α promoter before and after differentiation. EGFP expression was stably maintained in NSCs in long-term culture (Supplementary Figure S7B). To identify whether the change of chromosomal landscape affects transcriptional activity of EF1α promoter, we added 25 ng/ml IFN-γ for 48 h to iPSCs and NSCs. IFN-γ treatment did not affect EGFP expression in iPSCs or NSCs (Figure 3F). In contrast, surface β2m expression in NSCs was increased 19.8 ± 0.9-fold by IFN-γ treatment (Figure 3G). Therefore, silencing of transgene expression in NSCs occurs post-transcriptionally, rather than by alteration of promoter activity.

### The Autophagy Pathway Is Not Responsible for HLA-I Silencing

HLA-I expression could be restricted by the autophagy pathway and retained by the inhibition of lysosomal degradation (Yamamoto et al., 2020). Because the transcriptional activity was intact, we restored HLA-I expression using chloroquine (CQ), an autophagy inhibitor. After incubation with 50 μM CQ for 48 h, Non-treated control (NTC) cells showed intracellular β2m puncta, which were co-localized with LAMP-1 positive lysosomes (Supplementary Figure S8B). In CQ-treated cells, β2m and lysosome co-localization was disorganized but β2m localization was comparable with the untreated control. Furthermore, β2m surface expression was unaffected by CQ (Supplementary Figure S8A). In contrast, IFN-γ treatment of NSCs upregulated cell surface expression of β2m (Supplementary Figures S8A,B). Therefore, the suppression of HLA-I expression was not caused by transcriptional changes nor by intracellular protein recycling; instead, it was caused by other genes involved in HLA-I processing and presentation.

### Downregulation of Antigen-Presentation Machinery Genes Restricts Transgene Expression in NSCs

**FIGURE 4:**
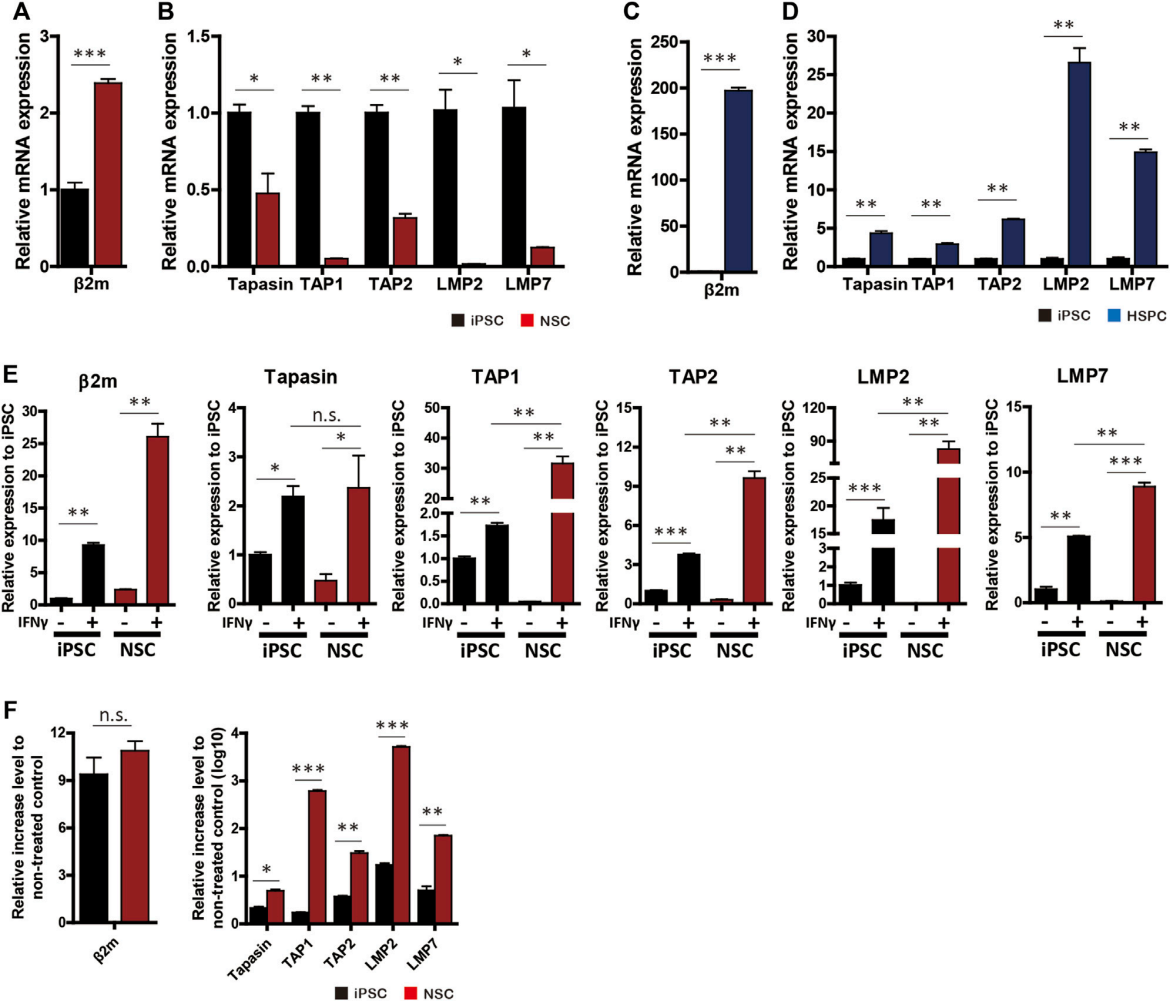
APM gene expression profiles. **(A–D)** mRNA levels of β2m and APM genes (tapasin, TAP1, TAP2, LMP2, and LMP7) in neural-lineage cells **(A,B)** and hematopoietic-lineage cells **(C,D)** in comparison with iPSCs (*n* = 4). **(E)** β2m and APM expression patterns in iPSCs and NSCs in response to 48 h of IFN-γ treatment (*n* = 4). **(F)** Rate of increase in gene expression after IFN-γ treatment (*n* = 4). Data are means ± SEMs. n.s., not significant. *p < 0.05, **p < 0.01, ***p < 0.001.

To identify the cause of HLA-I and transgene silencing in NSCs, we investigated the expression patterns of β2m and APM genes. The β2m mRNA level in NSCs was significantly upregulated compared to iPSCs (mean 2.39 ± 0.09-fold increase), whereas NSCs did not express HLA-I on the surface (Figure 4A). Hematopoietic cells showed a mean 197.13 ± 5.65-fold increase in the β2m mRNA level compared to iPSCs (Figure 4C).

Next, we used RT-qPCR to measure the expression patterns of APM genes (tapasin, transporter associated with antigen processing [TAP] 1, TAP2, large multifunctional proteasome subunit [LMP] 2, and LMP7) in iPSCs, NSCs, and HPCs with or without IFN-γ. All APM genes were significantly downregulated in NSCs compared to iPSCs (Figure 4B). In contrast, HPCs showed significantly higher APM gene expression than did the other cell lines (Figure 4D).

Since β2m expression was upregulated and translocated to the surface in NSCs in response to IFN-γ, we compared β2m and APM expression patterns before and after IFN-γ treatment. β2m and APM were significantly upregulated by IFN-γ treatment in iPSCs and NSCs (Figure 4E). In the IFN-γ-treated groups, the expression levels of all APM genes except tapasin were significantly higher in NSCs than iPSCs (Figure 4E). The rate of increase in APM gene expression was significantly higher in NSCs, whereas the rate of increase in β2m gene expression did not significantly differ (Figure 4F). Therefore, transgene silencing in hypoimmunogenic cells is attributable to the downregulation of the expression of APM genes and can be restored by the upregulation of such genes.

### Restoration of Human Leukocyte Antigen Surface Presentation by Overexpression of Transcription Factors

We overexpressed transcription factors responsible for inflammatory signal transduction and HLA-I expression. HLA-I expression is governed by the activator subunit of NF-κB heterodimer, RelA (Forloni et al., 2010), and the key transcriptional regulator of HLA-I, NLRC5 (Neerincx et al., 2012). A constitutively active form of NLRC5, which harbors a mutation in the nucleotide triphosphate hydrolysis motif (Meissner et al., 2010), was also used.

**FIGURE 5:**
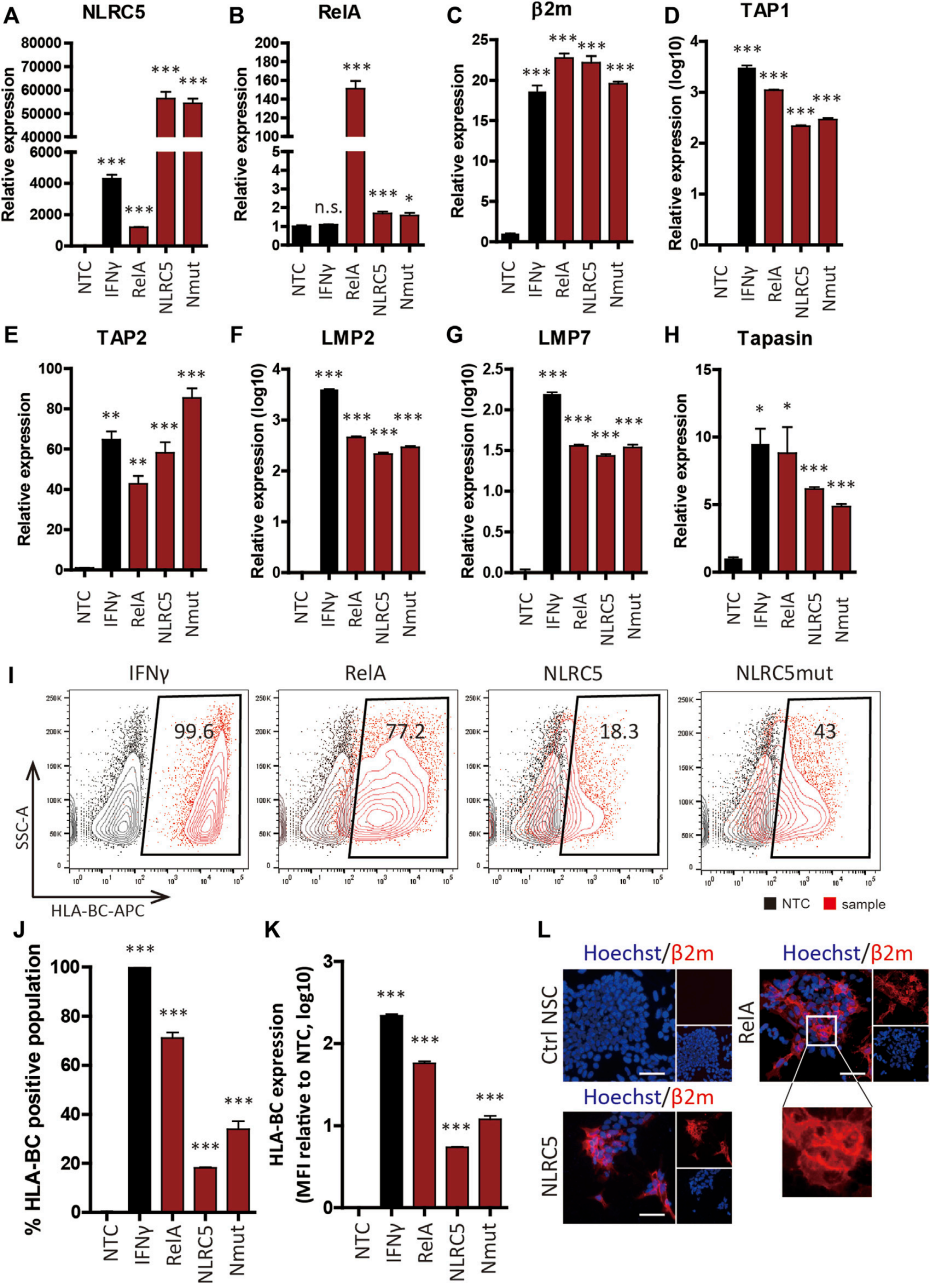
Upregulation of APM gene expression and HLA class I molecule surface presentation by transcription factors. **(A–H)** Relative mRNA levels in non-transfected control (NTC), IFN-γ-treated (IFN-γ), RelA-transfected (RelA), NLRC5-transfected (NLRC5), and constitutive mutant form of NLRC5-transfected (Nmut) NSCs (*n* = 4). **(I)** Representative flow cytometry plot of surface HLA-BC presentation. **(J,K)** Percentage HLA-BC-positive populations **(J)** and MFI values compared to NTCs **(K)**, (*n* = 4). **(L)** Immunocytochemistry of β2m surface presentation in response to transcription factors. Magnified image shows β2m on the cell surface; all images are fluorescence micrographs. Scale bar, 50 μm. Data are means ± SEMs.

RelA-, NLRC5-, and a constitutive mutant form of NLRC5 (NLRC5mut)-overexpressing plasmids were constructed and transiently transfected into cells; overexpression of NLRC5 and RelA was successful (Figures 5A,B), leading to significant upregulation of β2m and APM genes (Figures 5C–H). Some genes were upregulated in a manner similar to the pattern induced by IFN-γ. Flow cytometry showed that surface expression of HLA-I was significantly upregulated in NSCs at 48 h post-transfection (Figure 5I). RelA caused the greatest increase (57.8 ± 6.96-fold) in HLA-I surface presentation compared to the non-treated control; 71.05 ± 4.47% of the cells expressed HLA-BC. NLRC5 and NLRC5mut increased the MFI value 5.43 ± 0.17 and 12.13 ± 2.67-fold, respectively; 18.175 ± 0.39% and 33.9 ± 6.56% of the cells expressed HLA-BC (Figures 5J,K). Fluorescence imaging showed correctly localized HLA-I molecules on the cell surface (Figure 5L). Therefore, overexpression of transcription factors induced surface expression of HLA-I by regulating APM genes.

### NLR-Family CARD Domain-Containing 5 Recovers Transgene Expression and Hypoimmunogenicity of Gene-Edited Cell Lines

**FIGURE 6:**
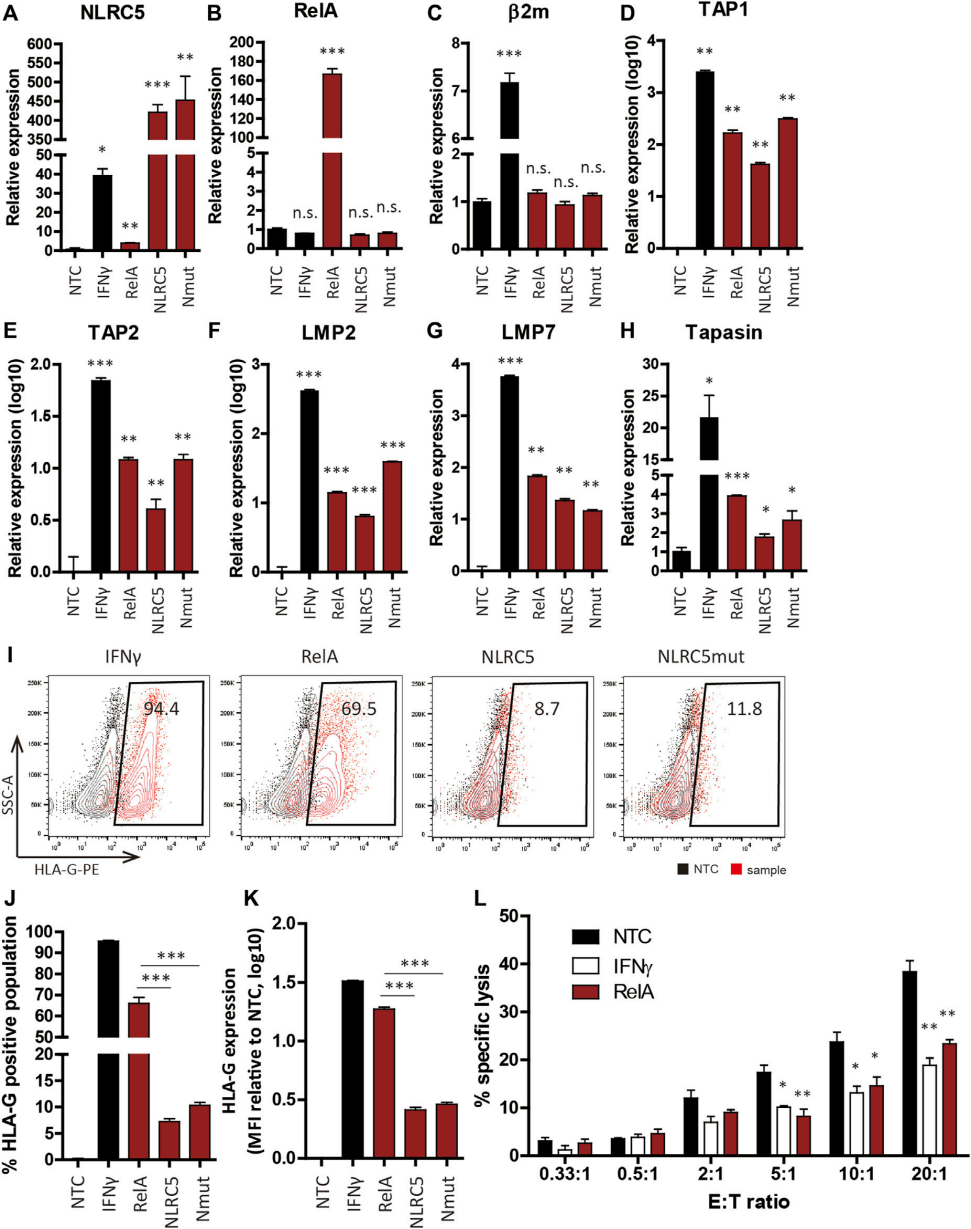
Transcription factors recovered HLA-G expression in hypoimmunogenic cell lines. **(A–H)** mRNA levels in non-transfected control (NTC), IFN-γ-treated (IFN-γ), RelA-transfected (RelA), NLRC5-transfected (NLRC5), and constitutive mutant form of NLRC5-transfected (Nmut) NSCs derived from hypoimmunogenic iPSCs (*n* = 4). **(I)** Representative flow cytometry plot of HLA-G surface presentation. **(J,K)** Percentage HLA-G-positive populations (J) and mean MFI values (**(K)**, *n* = 4). **(L)** Percentages of NK cell-specific lysis in NTCs (black), IFN-γ-treated (white), and RelA-transfected (red) cells were assessed at the indicated effector to target ratios (E:T ratios). Data means ± SEMs. *p < 0.05, **p < 0.01, ***p < 0.001.

To assess whether NLRC5 overexpression could recover transgene expression in gene-edited cell lines, we overexpressed RelA, NLRC5, and NLRC5mut in NPCs derived from gene-edited cell lines. At 48 h post-transfection, overexpression of the three transcription factors significantly upregulated the transcript levels of APM genes; meanwhile, the transcript level of β2m-HLA-G was unaffected because its expression is controlled by the EF1α promoter (Figures 6A–H). In IFN-γ-treated cells, however, the β2m transcript level was significantly increased compared to NTCs or transfected cells (Figure 6C). Although the underlying mechanism is unclear, we surmise that IFN-γ may affect the stability of the β2m-HLA-G transcript. IFN-γ upregulates the transcript level of HLA-A2 by stabilizing mRNA (Snyder et al., 2001). Flow cytometry showed that transfected cells presented HLA-G on the cell surface and notably, RelA promoted significantly greater upregulation of HLA-G presentation compared to NLRC5 and NLRC5mut (Figures 6I–K).

To assess the immune-inhibitory potential of transfected cells, we performed calcein-AM release assays using NK92 cells. Since non-transfected control (NTC) NSCs do not present HLA-I on the surface, we hypothesized that they would be vulnerable to NK cell-mediated killing, whereas HLA-G-recovered NSCs would not. As expected, NK92 cell-mediated lysis was significantly diminished in RelA-transfected cells compared to NTCs (Figure 6L). Therefore, our strategy promoted retention of the hypoimmunogenicity of NSCs derived from gene-edited cell lines by modulating the expression of APM genes, thereby protecting NSCs from NK cell-mediated killing.

## Discussion

In this study, we showed that the hypoimmunogenic properties of gene-edited cell lines can be disrupted by epigenetic factors that arose during differentiation into functional derivatives and can be retained by overexpression of the transcription factors, RelA and NLRC5. Previously, Gornalusse et al. (2017) and Han et al. (2019) used HLA-E and HLA-G to generate universally acceptable hypoimmunogenic PSCs; in both studies, depletion of immunogenic molecules and overexpression of immune-inhibitory molecules rendered cells hypoimmunogenic. These findings demonstrate the possibility of manufacturing off-the-shelf stem cell therapeutics. Our findings indicate that unless the genetically modified hypoimmunogenic cells utilize molecules belonging to the HLA-I gene family, therapeutic translation of them could be hindered by suppression of immune-inhibitory gene expression. The use of CD47 (Deuse et al., 2019), also known as the “do not eat me” signal, could overcome this issue (Van et al., 2006; Lehrman et al., 2018). However, because the inhibitory receptor profile differs among NK cell subsets, other immune-inhibitory genes may be linked to the evasion of innate immunity. For this reason, further work is needed to enhance the utility of hypoimmunogenic cells.

To generate hypoimmunogenic cells, we evaluated the surface expression and immunomodulatory function of a β2m-HLA-G fusion construct. K562 cells with surface HLA-G were protected against NK92-cell-mediated lysis. To create hypoimmunogenic iPSC lines, we induced homology-directed repair by using CRISPR/Cas9 to knock-out the β2m gene and knock-in the HLA-G in a single step. Although we did not create a biallelic cell line, we established cell lines that harbored a transgene at the β2m locus and indel mutations on the other strand. These cell lines showed no HLA-I surface expression even after stimulation with IFN-γ but constitutively presented HLA-G on the surface.

The hypoimmunogenic PSCs were differentiated into hematopoietic and neural cells. The expression trends in neural cells were distinct from the trends in pluripotent or hematopoietic cells. The expression trend in hematopoietic cells was similar to the trend in pluripotent cells, but neural cells did not have surface HLA-G despite the transcriptional activity of the EF1α promoter being unperturbed. HLA-I expression can be suppressed by the autophagy pathway (Yamamoto et al., 2020), transcriptional silencing (van den Elsen, 2011; Ramsuran et al., 2015; Kulkarni et al., 2017), and downregulated APM genes (Suarez-Alvarez et al., 2010; Ritter et al., 2017). In this study, we found that decreased expression of APM genes was responsible for HLA-I suppression in neural cells. The transcript level of β2m was higher in neural cells than in pluripotent cells; only APM genes were globally downregulated to an almost-undetectable level in neural cells. Previous articles addressed the altered expression of HLA-I, β2m or APM genes during differentiation into hematopoietic cells (Basak et al., 2009; Sabir et al., 2013), embryoid bodies (Cabrera et al., 2007; Suarez-Alvarez et al., 2010), neural crest cells (Mehler et al., 2020), osteoblasts and adipocytes (Sabir et al., 2013). In this article, we demonstrated that transcripts of β2m emerged upon differentiation induction of hPSCs. However, to date, no causal evidence has been presented to suggest an incomplete translocation of HLA-I into the membrane, as opposed to the increased expression of β2m, which is a result of APM regulation leading to the observed changes in immunogenic features of differentiated cells, especially in neural lineage. Although the exact underlying mechanism has not been fully elucidated, β2m, in addition to the small chain of HLA-I, displayed a variety of physiological roles such as a precursor of antibacterial chemokine (Chiou et al., 2016), a systemic pro-aging factor impairing cognitive function (Smith et al., 2015), and a diagnostic factor in several diseases (McArthur et al., 1992; Brew et al., 1996; Corlin et al., 2005). Therefore, it is possible to consider that different regulatory mechanisms independent of APM or HLA-I may account for β2m expression. Further studies will be required to clarify how the transcripts of β2m are upregulated even APM is suppressed and the different physiological roles of β2m in neural lineages.

Since APM genes were critical for HLA-G surface presentation, we investigated a method for restoring their expression in a cell-autonomous manner to avoid unwanted systemic effects. IFN-γ upregulates APM genes and HLA-G surface expression; however, since it works systemically, it is unsuitable as a therapeutic option. Overexpression of the HLA-I regulators, RelA and NLRC5, induced HLA-I surface presentation by upregulating APM gene expression. Both RelA and NLRC5 significantly promoted HLA presentation in WT NSCs. In gene-edited cells, NLRC5 upregulated the transcript level in a manner similar to the effect of RelA; however, the effect of RelA was significantly greater than the effect of NLRC5. The underlying mechanism is unclear but may be related to the HLA transcription control system. Unlike WT genes, HLA-G expression is controlled by the EF1α promoter, which is unaffected by RelA or NLRC5. Since NLRC5 regulates APM genes along with HLA-I and β2m (Meissner et al., 2010), its effect could be altered in gene-edited cell lines. Additionally, NLRC5 suppresses the inflammatory response and inhibits the NF-κB signaling pathway (Benko et al., 2010; Cui et al., 2010; Mu et al., 2021); NLRC5 overexpression inhibits inflammation by activating the autophagy pathway in human ectopic endometrial stromal cells (He et al., 2020). A notable limitation of our transient transfection approach was that variations in transfection efficiency may have affected the results. Although we generated hypoimmunogenic cells with considerable potential for therapeutic translation from HLA-I active to silenced cells, stable cell lines are needed. Furthermore, there are potential disadvantages associated with consistent expressions of our candidate genes, such as cell death (Collett and Campbell, 2006; Block et al., 2007) and cell cycle arrest (Sheehy and Schlissel, 1999). Genes participating in the IFN-γ signaling cascade (e.g., JAK1, JAK2, STAT1, STAT3, STAT6, etc) would be good alternatives to RelA or NLRC5. The interferon regulatory factor 1 (IRF-1), which is responsible for signal transduction of IFN-γ, is known to promote β2m transcription via directly binding to the interferon-stimulated response element (ISRE) (Gobin et al., 1999). Enhanced HLA-I expression was mediated by binding of IRF-1 to ISRE and binding nuclear accumulate p65 to enhancer A elements located in promoter region (Shen et al., 2009; Lorenzi et al., 2012). In addition, ectopic expression of IRF1 did not display any toxic effect on the normal primary cells such as HUVEC (Lee et al., 2008). In this respect, we believe that IRF1 will be one of the good candidates to enhance hypoimmunogenic activity.

In addition, the safety issues associated with the neoplastic transformation of hPSC-derived cells are a crucial prerequisite for the development of cell-based therapies. This is especially important because hypoimmunogenic cells are intentionally designed to escape the host’s immune system. To remove potentially harmful cells, suicide programs, prodrug-based systems such as herpes simplex virus thymidine kinase (HSVtk) gene and cytosine deaminase (CD) gene linked with ganciclovir (GCV) and 5-fluorocytosine (5-FC), respectively, have been developed (Jones et al., 2014). Recently, an inducible caspase 9 (iCasp9) system in which genetically modified caspase 9 is fused to FK506 binding protein (FKBP) has been attempted to eliminate inappropriately activated cells (Straathof et al., 2005; Gargett and Brown, 2014). Numerous groups have validated the stability, efficacy, and safety of suicide systems *in vitro* and *in vivo* (Yagyu et al., 2015; Liu et al., 2022), but the evaluation of the safety level including the type/number of cells and the type of genetic modification should be further considered (Liang et al., 2018). In order to improve the safety of cell therapy products, it is necessary to apply a safety system along with the development of hypoimmunogenic cells.

Finally, we believe our method will promote the development of cell replacement therapies using hypoimmunogenic donor cells and ultimately facilitate the manufacture of off-the-shelf hypoimmunogenic donor cell products.

## Data Availability

The original contributions presented in the study are included in the article/Supplementary Material; further inquiries can be directed to the corresponding author.

## Supplementary Material

Supplementary Figure S1 cDNA. sequence of β2m-linker-HLA-G. β2m cDNA sequence with a silent mutation to avoid off-target effects (PAM mut) is directly linked to the HLA-G cDNA lacking a leader sequence using a Gly-Gly-Gly-Gly-Ser-Gly-Gly-Gly-Gly-Ser-Gly-Gly-Gly-Gly-Ser-Gly-Gly-Gly-Gly-Ser (GGGGS)4 flexible protein linker. Silent point mutations in the HLA-G cDNA sequence are in red.

Supplementary Figure S2
*In vivo* teratoma formation assay of the iPSC line. Characterization of the differentiation potential of the iPSC line used to generate gene-edited hypoimmunogenic cell lines. Their differentiation capacity was examined by *in vivo* teratoma formation assay. H&E images, from left, ectoderm-derived pigmented cells, mesoderm-derived cartilage, and glands from endoderm lineage cells. White arrowheads depict differentiated cell types in teratoma. Magnification × 40. Scale bar, 200 μm.

Supplementary Figure S3 Characterization of β2m KO cell lines using western blot and immunocytochemistry. **(A)** Lane1: lysate of control iPSC (CTRL iPSC), Lane2-5: lysate of b2m KO cell lines (the number of cell lines is indicated at each lane), GAPDH as housekeeping gene. **(B)** Immunofluorescence micrographs against b2m (red) and nucleus (Hoechst 33342, blue). The representative images were taken in control iPSC colony (Ctrl iPSC) and the b2m KO cell line (b2m KO #A16). Scale bar, 250 μm.

Supplementary Figure S4 Off-target analysis. **(A)** gRNA sequence used for cell line construction and the protospacer adjacent motif (PAM) sequence (red). Predicted all-exonic off-target sites; mismatched sequences are in small red letters. **(B)** Reference sequences of 10 exonic off-target sites from NCBI (reference seq, GRCh38.p13) and sequences of wild-type (Ctrl iPSC) and hypoimmunogenic (A5 and A9) cell lines.

Supplementary Figure S5 Differentiation of iPSCs into hematopoietic and neural lineages. **(A)** Schematic of hematopoietic and neural differentiation, with representative light micrographs. **(B,C)** Immunofluorescence micrographs of neural stem cell markers **(B)** and a neuronal marker **(C)**. Nestin (green), Sox (red), Tuj1 (green), and nucleus (Hoechst 33342, blue) were stained. **(D)** Flow cytometry data for hematopoietic stem cell markers in cells differentiated from WT (control), β2m knock-out (β2m KO), and hypoimmunogenic (A5 and A9) iPSCs. Scale bar, 250 μm.

Supplementary Figure S6 Flow cytometry analysis of HLA-I expression in response to IFN-γ treatment and withdrawal. **(A)** HLA-BC and HLA-G expression patterns in wild-type (Ctrl NSC) and gene-edited (A9 NSC) NSCs under normal conditions (non-treated), after IFN-γ treatment (48 h IFN-γ), and 3, 9, and 13 days after IFN-γ withdrawal (w/o IFN-γ D+3, +9, and +13). **(B)** Relative MFI values at the indicated timepoints (*n* = 3).

Supplementary Figure S7 EGFP cell line generation and characterization before and after differentiation. **(A)** Schematic of the HR donor template plasmid for EGFP cell line generation. **(B)** EGFP expression in pluripotent cells (iPSC) and neural cells (NSC) differentiated from the wild type (Ctrl) and (EGFP) EGFP cell lines.

Supplementary Figure S8 HLA-G expression patterns in response to lysosomal inhibitors. **(A)** HLA-G expression in the non-treated control (NTC) and after 48 h of 25 ng/ml IFN-γ (IFN-γ) and 50 μM chloroquine (CQ) treatment. **(B)** Representative confocal micrographs of NSCs. Nuclei were stained with Hoechst 33342 (blue). HLA-G was stained with β2m and a secondary antibody (red), and lysosomes were stained using an APC-conjugated LAMP-1 antibody (cyan). Scale bar, 50 μm.
