# Supplementary figures and images for: Maintenance of Hypoimmunogenic Features via Regulation of Endogenous Antigen Processing and Presentation Machinery
Source: Front Bioeng Biotechnol. 2022 Jul 22;10:936584. doi: 10.3389/fbioe.2022.936584 (PMC9416868; doi:10.3389/fbioe.2022.936584)

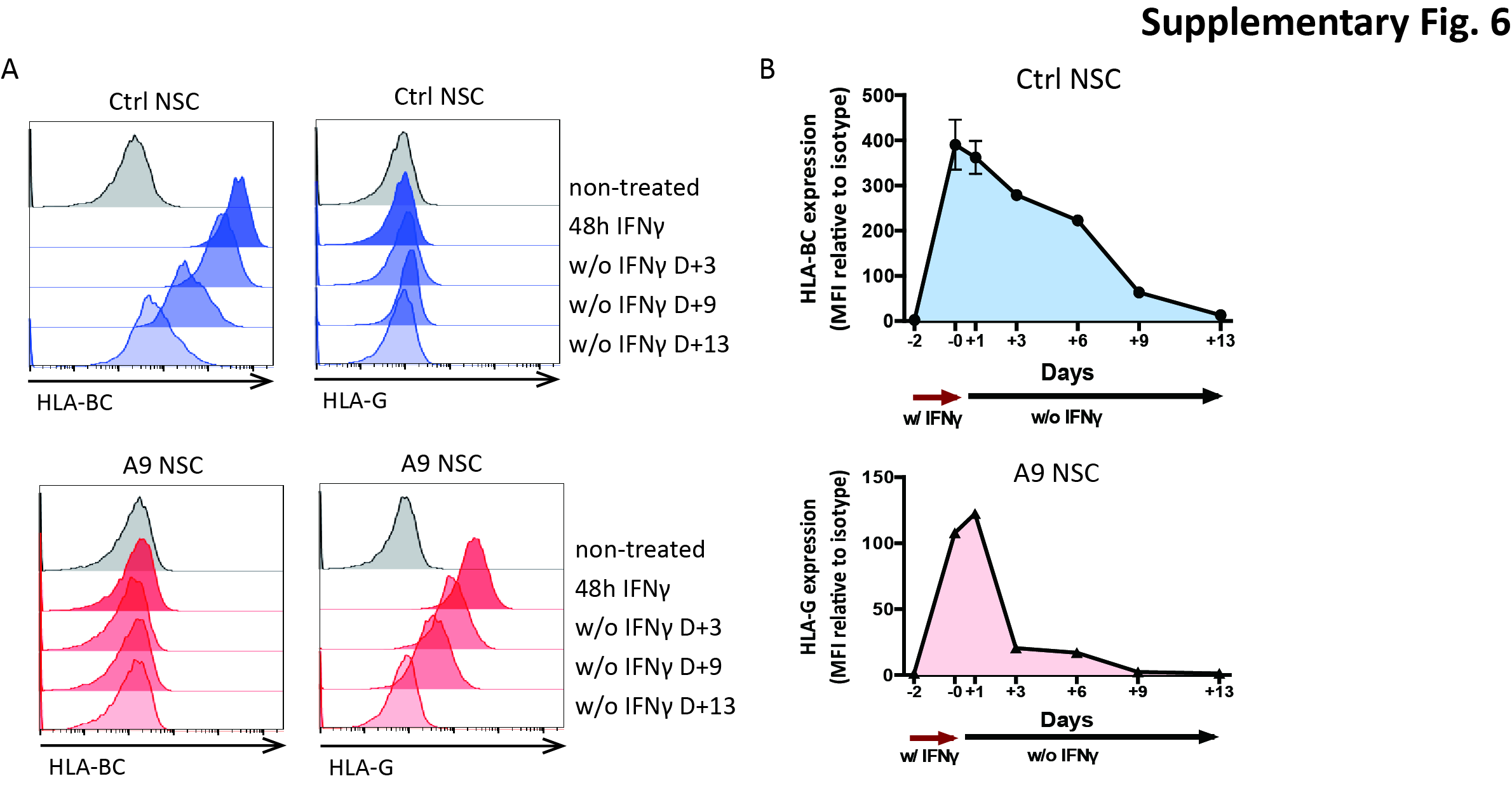

Supplement: Supplementary file 1 [file Image6.TIF]

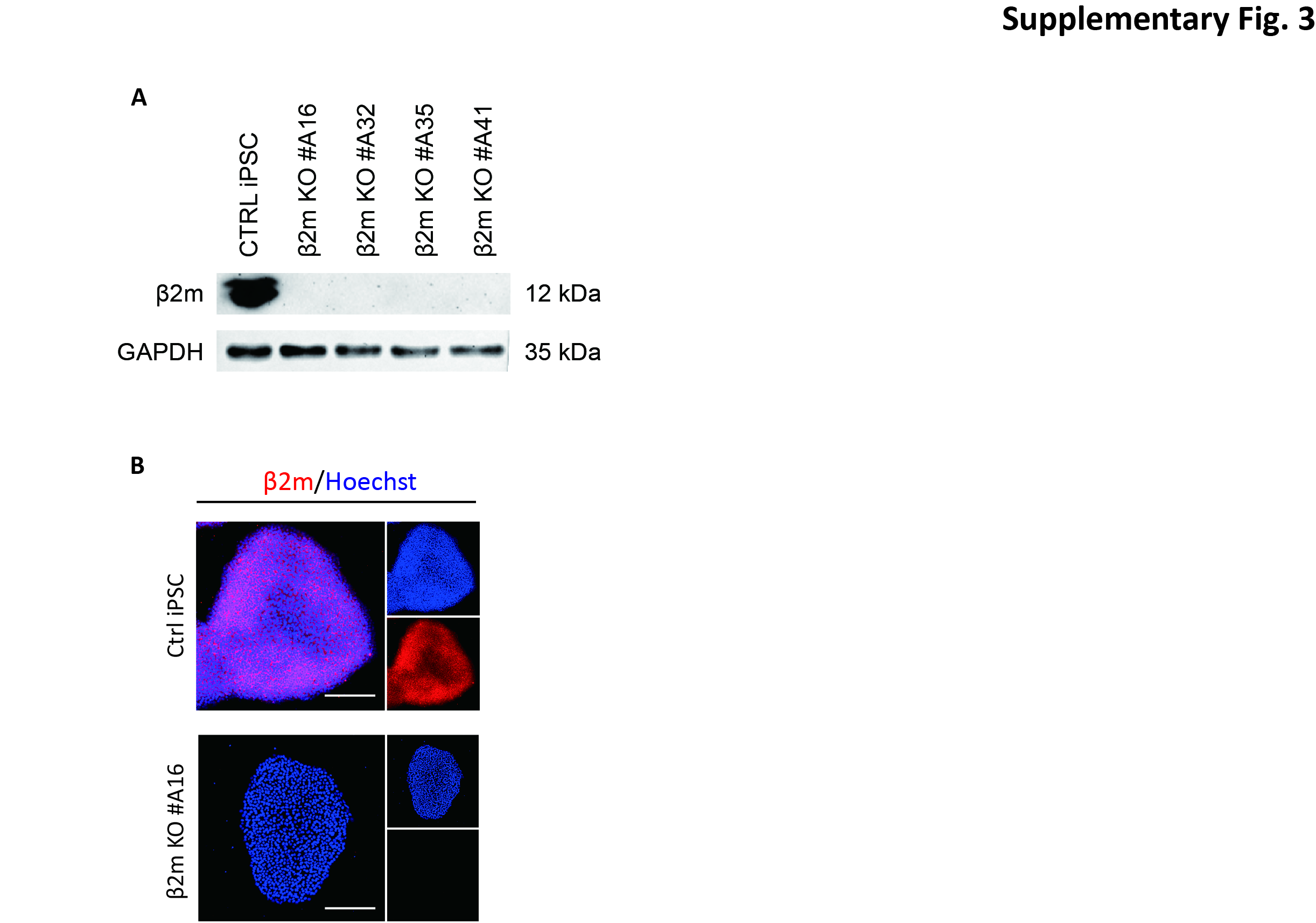

Supplement: Supplementary file 3 [file Image3.TIF]

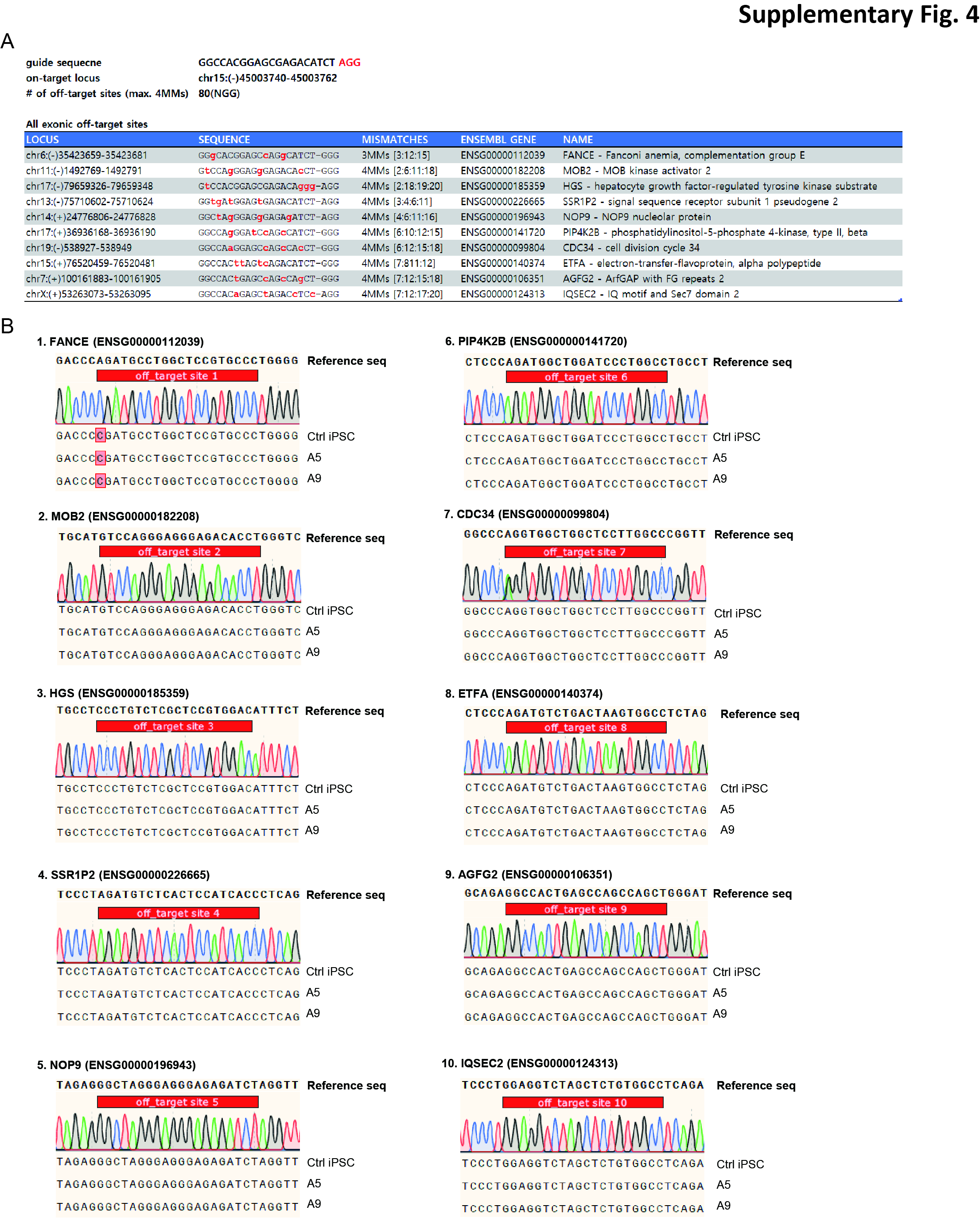

Supplement: Supplementary file 4 [file Image4.TIF]

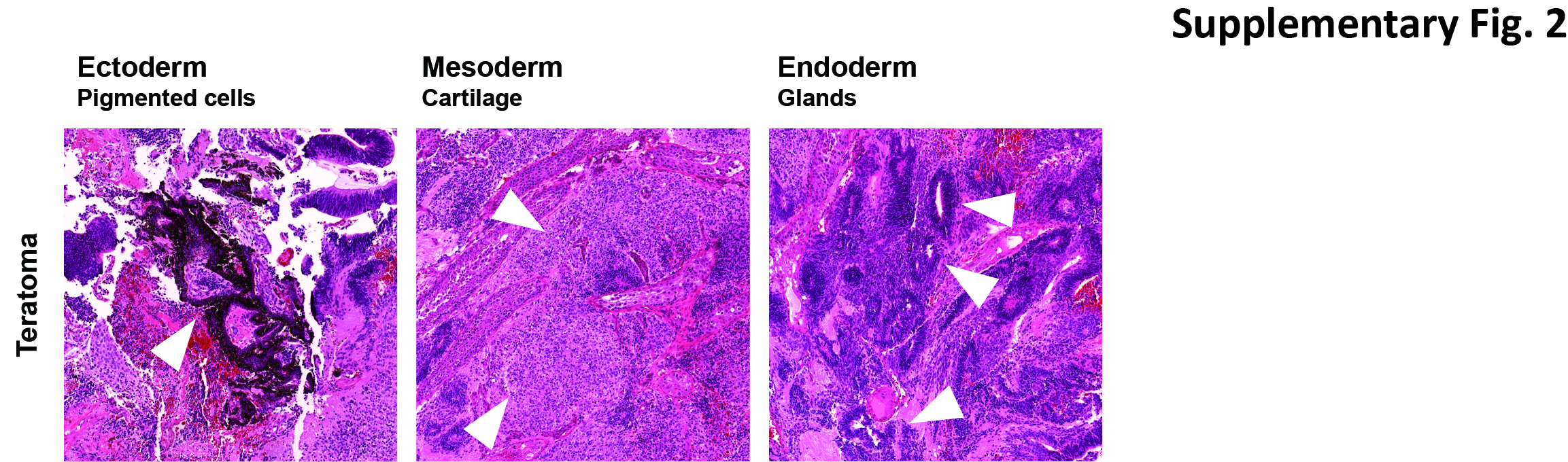

Supplement: Supplementary file 5 [file Image2.TIF]

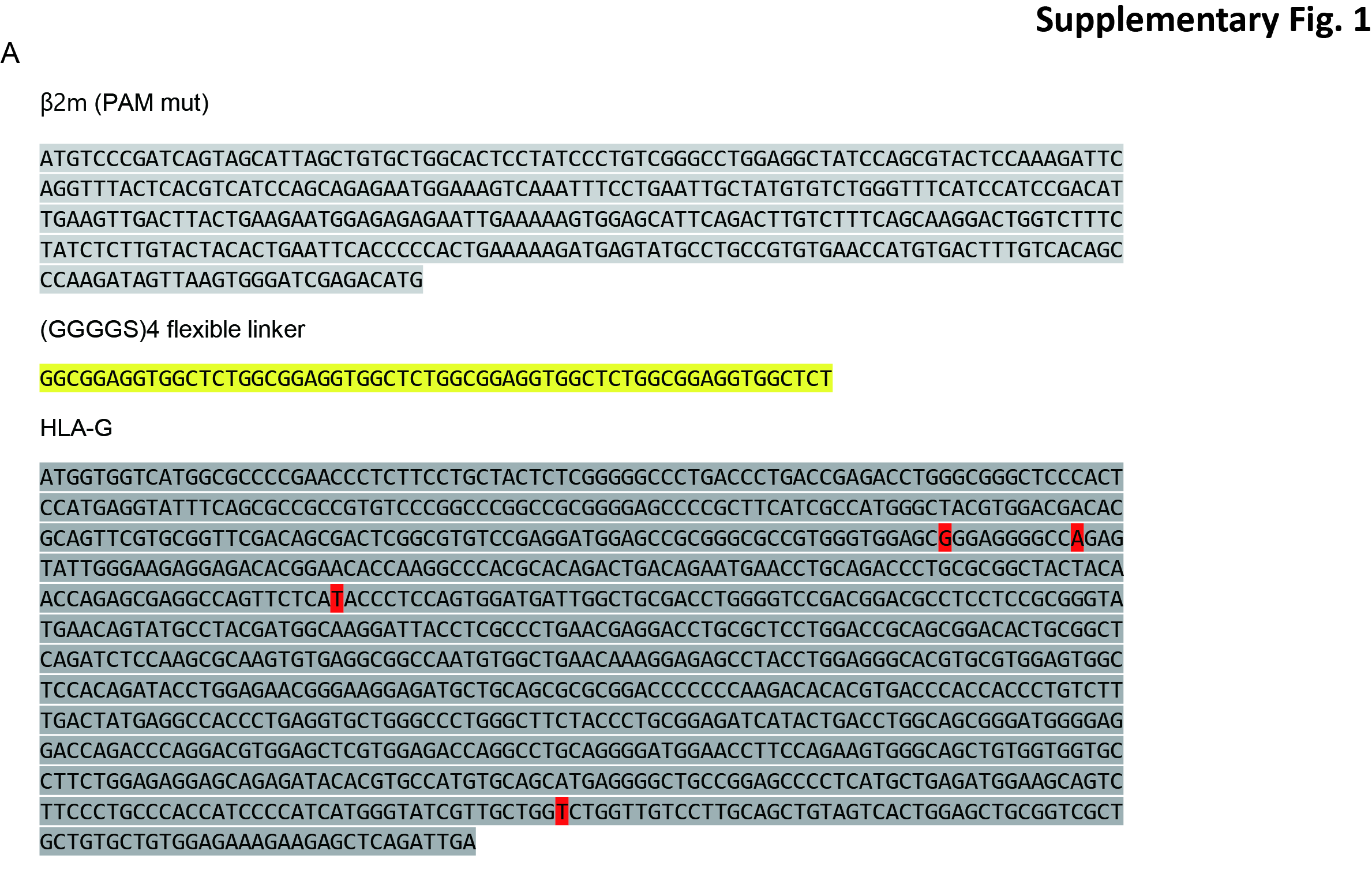

Supplement: Supplementary file 6 [file Image1.TIF]

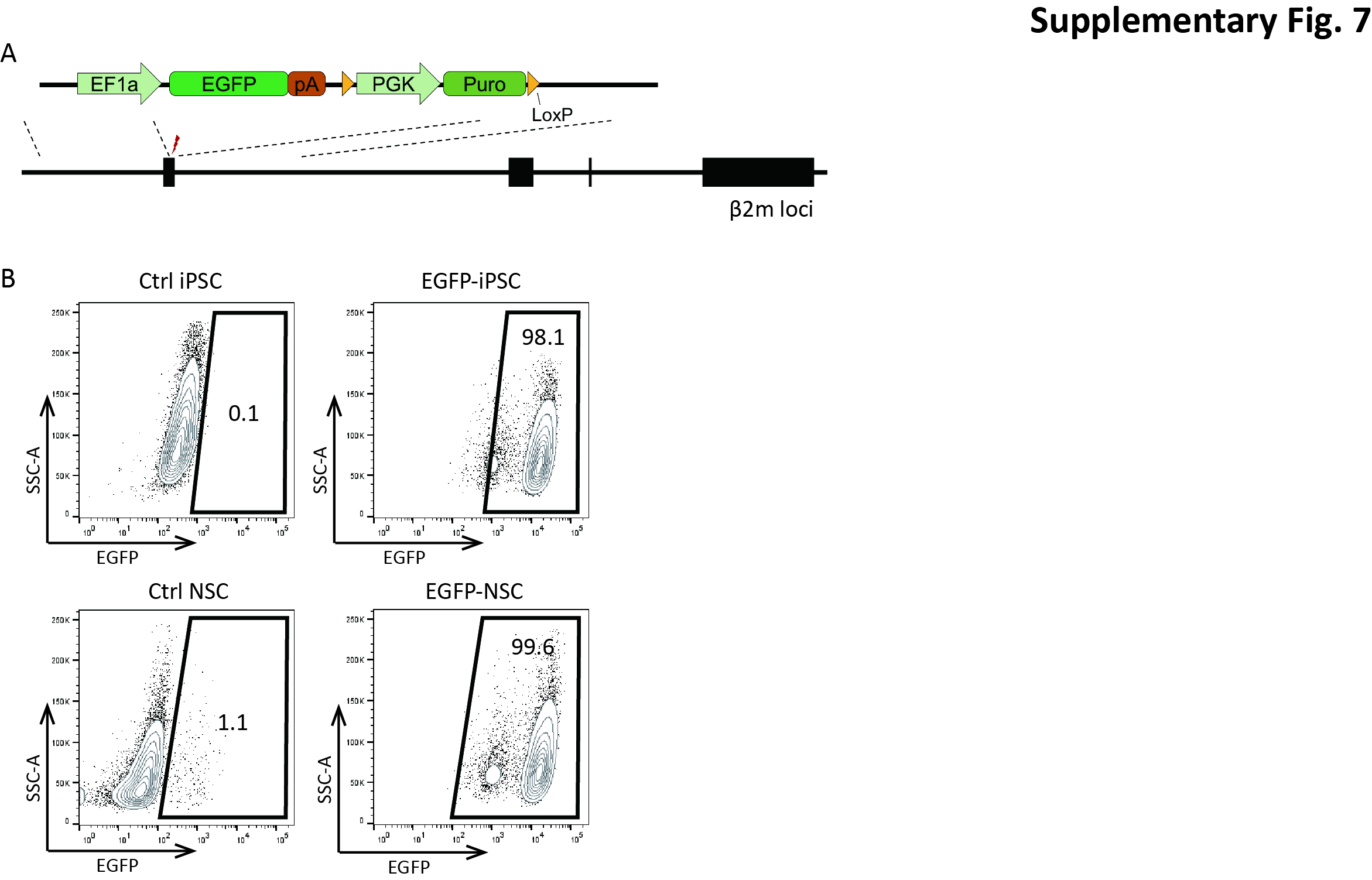

Supplement: Supplementary file 7 [file Image7.TIF]

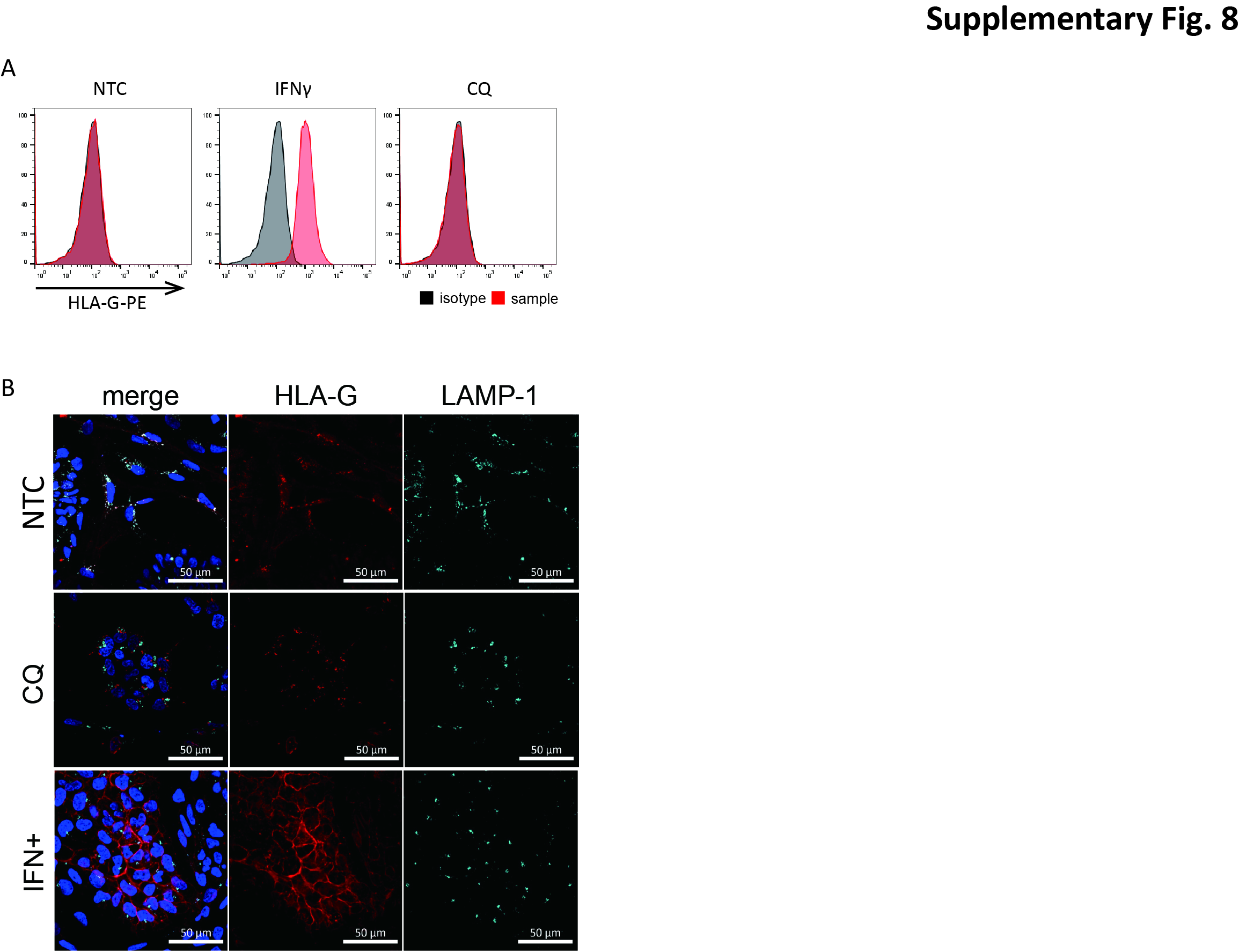

Supplement: Supplementary file 8 [file Image8.TIF]

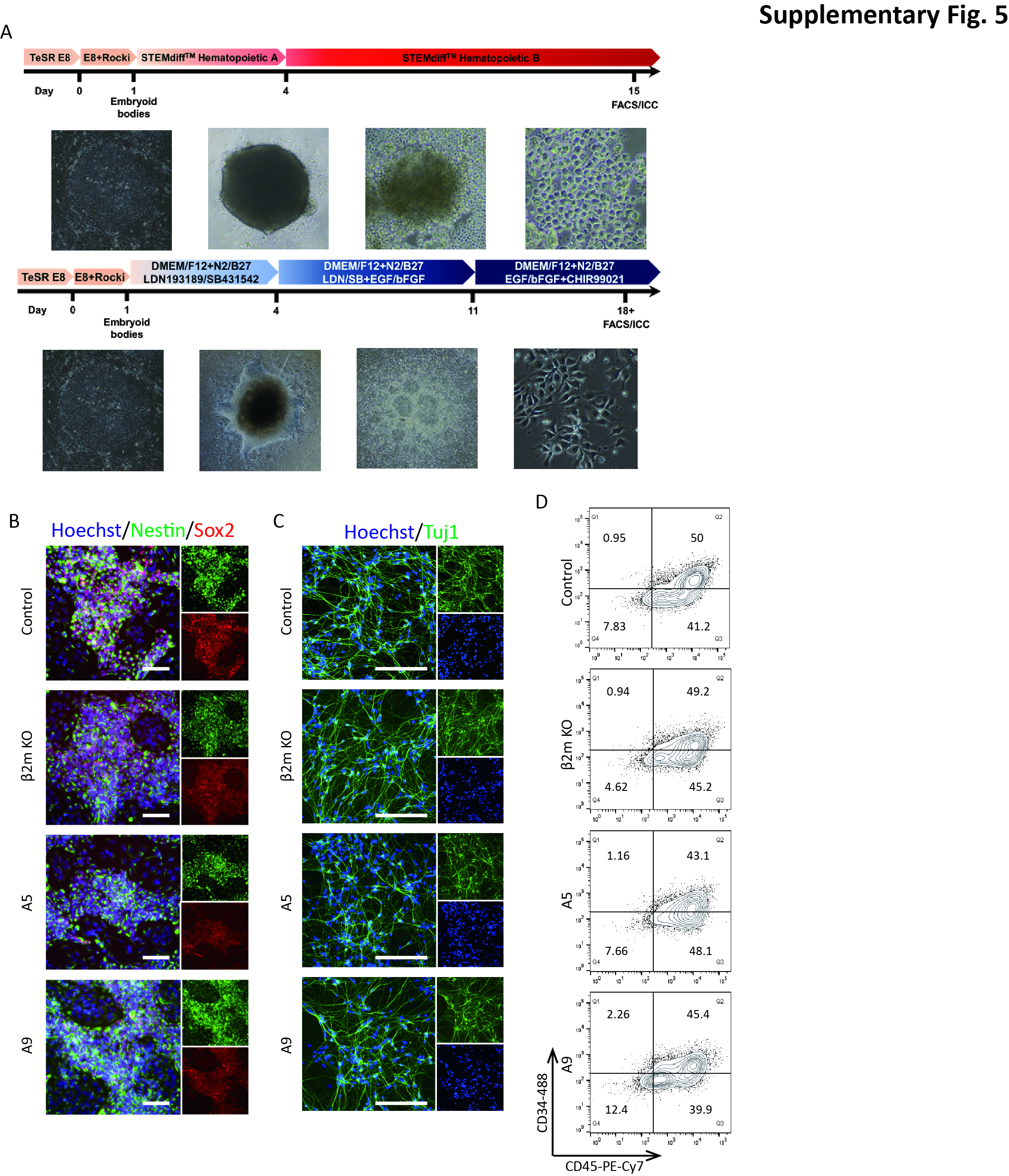

Supplement: Supplementary file 9 [file Image5.TIF]
