# Supplementary material for: Maintenance of Hypoimmunogenic Features via Regulation of Endogenous Antigen Processing and Presentation Machinery
Source: Front Bioeng Biotechnol. 2022 Jul 22;10:936584. doi: 10.3389/fbioe.2022.936584 (PMC9416868; doi:10.3389/fbioe.2022.936584)
Supplement: Supplementary file 2 [file Table1.DOCX]

| **Supplementary Table S1.** Primers used for each experiments. | |
| --- | --- |
|  |  |
| **Primer Name** | **Sequence (5' to 3')** |
| **Genotyping and indel analysis** | |
| b2m_seq_F | CCTCCAGCCTGAAGTCCTAGAATGAGC |
| b2m_seq_R | CGGCGCTCTGACGCTTATCGACGCCC |
| **Real time quantitative PCR** | |
| HLA-A_RT_F | TCCTTGGAGCTGTGATCGCT |
| HLA-A_RT_R | AAGGGCAGGAACAACTCTTG |
| HLA-B_RT_F | TCCTAGCAGTTGTGGTCATC |
| HLA-B_RT_R | TCAAGCTGTGAGAGACACAT |
| HLA-C_RT_F | TCCTGGTTGTCCTAGCTGTC |
| HLA-C_RT_R | CAGGCTTTACAAGTGATGAG |
| HLA-G_RT_F | AGC TGT GGT GGT GCC TTC |
| HLA-G_RT_R | GGG CAG GGA AGA CTG CTT |
| TAP1_RT_F | CGCCTCACTGACTGGATTCTAC |
| TAP1_RT_R | TCCGTCTCCTGGCGCAGGAC |
| TAP2_RT_F | GTCGTGTCATTGACATCCTG |
| TAP2_RT_R | TCAGCTCCCCTGTCTTAGTC |
| Tapasin_RT_F | AGTGTACACGACCCCGCGGG |
| Tapasin_RT_R | CAGGCCGCTGGCCCATTTCG |
| LMP2_RT_F | TTGTGATGGGTTCTGATTCCCG |
| LMP2_RT_R | CCATGTCGGCCACGGCTTGGG |
| LMP7_RT_F | TCGCCTTCAAGTTCCAGCATGG |
| LMP7_RT_R | GCTGCACAGCCAGACATGGTGCC |
| NLRC5_RT_F | CTGGCCAGTCTCACCGCACAA |
| NLRC5_RT_R | CCAGGGGACAGCCATCAAAATC |
| RelA_RT_F | GCACAGATACCACCAAGACC |
| RelA_RT_R | GATGGCTTCTATGAGGCTGA |
| **Off-target sequencing** | |
| off_1_F | ACTCTTGCAGATACTGCGTGCCAGCC |
| off_1_R | TCAGGACTGGCAGCCTCTTCCTCTG |
| off_2_F | ACAGCAGCACGCACTTGTGTAAACG |
| off_2_R | CACTGTGAGGATGACCGCCCAGTGC |
| off_3_F | CCCTTGGCACTCAGATCTTCGCCTG |
| off_3_R | AGCCGCTGGCTCACAGCAGACTCAC |
| off_4_F | TATATTGCCTGCCACCCTCTTGTTC |
| off_4_R | TCATGCTTAAAGGGTCATTCACCCG |
| off_5_F | CTTACTTTGGCTAGCAGCTCCTGGC |
| off_5_R | TCAGCTCTGAACCCCAGTGACTTAC |
| off_6_F | CCGCCCCGTTTCTTCTGAAAGAGGC |
| off_6_R | TTGGCATGGCAGAGGGAGAGGCAAG |
| off_7_F | AGCATCCAGGGCACCTTCCATCCTG |
| off_7_R | CTCCACGGCAGCCTGCCCGACACAG |
| off_8_F | TGAAAGAATTATCTGAGCATGGTGG |
| off_8_R | AACATGGTATAGTCCAGGTAACTGC |
| off_9_F | AGCCCATGCAGTGCCCTTTCTGACC |
| off_9_R | GCTGGAACAGCATGGGAGATGCCAG |
| off_10_F | CATGTCCTCCCTGCCTTGGGACCCG |
| off_10_R | CCACGTGGGGCCAACACAAGCAGGG |
